# Navigating Informal Support in Regional Dementia Care Network: A Qualitative Exploration in Southeastern Germany

**DOI:** 10.1093/geroni/igaf122.2834

**Published:** 2025-12-31

**Authors:** Zi Yan, Shuji Tsuda, Kae Ito, Shuji Awata

**Affiliations:** Waseda University, Tokyo, Tokyo, Japan; Tokyo Metropolitan Institute of Geriatrics and Gerontology, Tokyo, Tokyo, Japan; Tokyo Metropolitan Institute of Geriatrics and Gerontology, Tokyo, Tokyo, Japan; Tokyo Metropolitan Institute of Geriatrics and Gerontology, Tokyo, Tokyo, Japan

## Abstract

Dementia Care Networks (DCNs) play a vital role in supporting persons with dementia (PWD) and their caregivers, yet informal support mechanisms within regional DCNs remain underexplored. This study explores informal support structures in regional DCNs in southeastern Germany, identifying facilitators and barriers for effective DCNs. An exploratory-descriptive qualitative approach was used, incorporating semi-structured interviews and participant observation with 23 individuals, including social workers, physicians, nurses, volunteers, family caregivers, and PWD across two Bavarian communities. Thematic analysis and structured content analysis, framed by the Social-Ecological Model (SEM), revealed three distinct forms of informal support that enhanced resilience for caregivers, PWD, and formal service providers. Facilitators spanned multiple SEM levels: heightened dementia awareness (individual), robust volunteer traditions (interpersonal), community-level social capital(community), collaborative networking platforms (organizational), and proactive local leadership (regional). A notable unanticipated finding was a spill-over effect, wherein informal support sustained community well-being and aided former caregivers post-bereavement. Conversely, sustainability challenges stemmed from organizational fragmentation, including limited stakeholder awareness and inter-stakeholder competition. The study underscores informal support’s critical role in mitigating pressures on formal care systems, particularly in resource-constrained regions. Findings offer actionable strategies for policymakers and practitioners to optimize DCN efficacy by leveraging community resources, fostering cross-sector collaboration, and addressing systemic barriers. This research contributes empirical insights to the discourse on dementia care, emphasizing the interplay of informal and formal support systems in promoting holistic, sustainable care models. Keywords Dementia, informal support; dementia care network, integrated care, care coordination, Germany

